# Validity of the Draw a Person: A Quantitative Scoring System (DAP:QSS) for Clinically Evaluating Intelligence

**DOI:** 10.1007/s10578-020-01058-6

**Published:** 2020-09-12

**Authors:** Alda Troncone, Antonietta Chianese, Alfonso Di Leva, Maddalena Grasso, Crescenzo Cascella

**Affiliations:** Department of Psychology, University of Campania “Luigi Vanvitelli”, Viale Ellittico 31, Caserta, Italy

**Keywords:** Intelligence, Draw a Person: A Quantitative Scoring System, Children, Adolescents, Scholastic achievement

## Abstract

To assess the psychometric properties of the Draw a Person: A Quantitative Scoring System (DAP:QSS), in 2543 children (*M* = 11.43 ± 3.06 years), correlations between drawings scores and Raven’s Matrices scores, age, and academic achievement were examined. Although older children (> 11 years) obtained higher drawing scores than younger ones (*p* < 0.001), age significantly correlated with DAP:QSS scores only in children younger than 11 years (*r* = 0.493, *p* < 0.001), indicating conflictive evidence for construct validity and a possible ceiling effect. No correlations emerged between DAP:QSS scores and grades (*r* = 0.056, *p* = 0*.*097). DAP:QSS scores were significantly associated with Raven’s Matrices score, but low correlation coefficients (0.156–0.498), low sensitivity (0.12), and high false negative (87.9%) and positive (82%) rates suggest poor DAP:QSS validity as an intelligence measure. The researchers concluded that DAP:QSS failed to produce a psychometrically sound assessment of children’s intellectual functioning.

The use and usefulness of human figure drawing tests as measures of intellectual ability have generated considerable debate [1–3]. Among the several methods of scoring human figure drawings, the Draw a Person: A Quantitative Scoring System (DAP:QSS) was developed by Naglieri in 1988 as an updated means of scoring the classic draw-a-person test. The DAP:QSS was designed for use with 5- to 17-year-olds as a nonverbal measure not influenced by linguistic variables that “can provide reliable information about intellectual status to supplement other intelligence test” [4, p. 2]. Created with reference to a well-normed standardization sample, the DAP:QSS uses three stimuli (i.e., drawings of a man, a woman, and the self) devised to avoid the possible confounding variables of fashion and dress.

Evidence suggests that the DAP:QSS generally yields high coefficients for intra- and inter-rater reliability [4–7], internal consistency, and test–retest reliability [4]. By contrast, the validity of the DAP:QSS as an instrument for assessing intelligence has yet to be satisfactorily demonstrated.

Moderate-to-high correlations with measures of verbal intelligence (e.g., Wechsler scales) [5, 8, 9] and nonverbal intelligence [4, 10–12], even among children and adolescents with learning disabilities [13], suggest that Naglieri’s DAP:QSS test can serve as a general screening measure of children’s and adolescents’ intelligence. Naglieri’s individual DAP drawings have also shown good correlations with the Goodenough–Harris’s drawing test [14, 15].

However, other researchers have found little support for the validity of the DAP:QSS as a tool for evaluating children’s intellectual ability and assessing children with mental disabilities [3, 16, 17]. Indeed, some researchers have not only concluded that the DAP:QSS is not a valid measure of intellectual ability and should not be used as a screening tool [7], but also suggested that human figure drawing tests should be eliminated from the repertoire of psychological assessment tools [18].

In examination of the utility of any measure of intelligence, relevant importance has been given to its ability to predict scholastic performance [3, 19]; consequently, the association between DAP:QSS and measures of academic achievement has also been examined, the results of which provide even more conflicting evidence. In particular, whereas some researchers found that the DAP:QSS cannot predict academic performance [3, 12, 17], others have described significant correlations between the DAP:QSS and scholastic achievement [4, 10, 20].

In recent investigations of the strengths and weaknesses of the DAP:QSS, researchers have analyzed whether DAP:QSS scores can be improved with practice [21], can be useful to identify highly gifted children [22], and are vulnerable to deliberate distortion by adolescents and young adults [23], due to generational changes (i.e., Flynn effect) [24], or due to the effects of gender [25]. In other work, Gentle, Powell, and Sharman [26] have examining the use of DAP:QSS as a protective exercise that lessens the impact of biased questions upon child witnesses. The DAP:QSS was also found to be specifically useful for evaluating cognitive and motor deficits of exceptionally preterm children [27].

Except for two studies that referred to an original normative sample of 2622 children aged 5–17 years in the United States [4, 28], studies geared toward analyzing the psychometric properties of the DAP:QSS have been conducted mostly with small samples of children with narrow age ranges in the United States ([5] *N* = 200, 6–15-year-olds; [7] *N* = 51, 6–16-year-olds), Canada ([11] *N* = 598, 6–10-year-olds), Greece ([14] *N* = 114 elementary-school children), Lithuania ([16] *N* = 165, 6–16-year-olds), Japan ([12] *N* = 400, 6–12-year-olds), New Zealand ([7] *N* = 125, 5–6-year-olds), and Italy ([15] *N* = 184 elementary-school children). Despite those studies, the psychometric properties of the DAP:QSS and its relationship with other commonly used measures of nonverbal ability need to be elucidated. As Abell et al. [5] have stated, “If they are to have any clinical validity, drawing tests must be examined with different population of children and different standard intelligence tests” [5, p. 206].

To address that need, the study presented here was conducted with a large sample of Italian children across a broad age range, with the aim of clarifying the psychometric properties of the DAP:QSS, especially in terms of its construct validity, concurrent validity, and usefulness in screening for intellectual difficulties. In particular, because construct validity can be determined by differentiating ability according to the child’s age [4, 29], the general developmental trend in DAP:QSS scores was analyzed as well. By contrast, concurrent validity was examined by comparing the DAP:QSS with more standard measures of nonverbal intelligence (i.e., the Raven Progressive Matrices).

Additionally, in order to simultaneously determine other aspects of the potential usefulness of the DAP:QSS, the relationship between children’s drawings and their academic achievement was examined for criterion-related validity by analyzing correlations between DAP:QSS scores and academic grades. Last, the usefulness of the DAP:QSS as screening tool for intellectual ability was investigated by verifying its ability to accurately identify children classified as having mental disabilities according to a valid measure of intelligence. Reliability tests—specifically, inter-rater and internal consistency coefficients calculation—were also conducted.

Specifically, the following four research questions were addressed.

1. What is the construct validity of the DAP:QSS?
2. What is the concurrent validity of the DAP:QSS and Raven’s Matrices with a group of individuals in the school-aged population?
3. What correlations exist between DAP:QSS and grades, and is it possible to predict children’s school achievement based on their DAP:QSS scores?
4. Is the DAP:QSS capable of discerning children who might be at risk for intellectual difficulties?

In examining those relationships, given evidence suggesting the significant influence of socioeconomic status (SES) on drawing performance [19], intelligence tests [30], and academic achievement [31, 32], SES was also taken into account. Similarly, in line with data describing gender differences in Naglieri’s drawings [12, 25, 27], boys’ and girls’ drawings were compared.

In accordance with the Naglieri’s assumption that DAP:QSS is a nonverbal intelligence measure [4], and with previous evidence supporting an association between DAP:QSS and measures of intelligence [4, 5, 8–13, 15] and scholastic achievement [4, 10, 20], this study hypothesizes that the Naglieri scoring system yields values that can be related to age, Progressive Matrices scores, and grades—but modestly, and not to the same extent that a more complex measure of IQ will. Similarly, given the poor evidence supporting DAP:QSS as an intelligence screening device [7, 17], it is expected that Naglieri’s drawings will show low accuracy in identifying children with low intellectual functioning.

## Method

### Participants

The study was conducted using a convenience sample of school-age children from local public primary, secondary, and high schools in five cities in Campania, Italy, who volunteered to participate. The schools were selected both on the basis of accessibility and to obtain roughly equal numbers of students from elementary, middle, and high school. A parent or guardian of each child who agreed to participate was asked to complete and sign a consent form. Students aged from 5 to 17 years who submitted signed parental permission forms indicating their consent to participate were included in the sample. Participants who needed an assistant teacher for a mental or physical disabilities were excluded, because due to the level, severity, and features of their disabilities, they would have probably requested specific adjustments in measurement procedures that were not entirely compatible with the assessment method or that would even negatively impact test administration (due to physical inaccessibility to rooms dedicated to evaluations, difficulties in moving, requiring presence of a special needs teacher, etc.). No extra credit was given for participation.

### Procedure

From October 2011 to October 2014, evaluations were made anonymously and collectively, with four children at a time distanced from each other to prevent peer influence, during class time in a room made available by the school and in the absence of the teacher. Participants were not told about the purpose of the study. Following the distribution of the normative sample age for Raven’s Colored Progressive Matrices (RCPM) [33], children aged from 5 to 11 years were evaluated with RCPM, whereas ones older than 11 years were evaluated with Raven’s Standard Progressive Matrices (RSPM). Both the RCPM and RSPM were administered without any time limit.

The students’ grades, when available, were obtained by directly consulting the students’ report cards. Academic achievement was evaluated as a mean of the grades obtained in six core subjects (i.e., Italian, history, geography, English, math, and science).

Parents were asked to complete a questionnaire addressing SES upon signing the consent form.

Four psychologists with master’s degrees adequately trained in the relevant techniques administered and scored all tests with reference to scoring instructions and the classification of intelligence included in the examiner’s manual for the DAP:QSS [4], RCPM and RSPM [33, 34]. The scoring of drawings was made without prior knowledge of the scores of the same subject for the RCPM or RSPM. The study was approved by the local ethics committee.

### Measures

#### Draw-a-Person: A Quantitative Scoring System (DAP:QSS)

The DAP:QSS [4] was developed to measure nonverbal aspects of intelligence in children 5–17 years old. Children are required to draw a picture of a man, a woman, and themselves. With an administration time of 5 min per drawing, the instrument can be administered individually or in a group. The system requires the application of the same 64 items to rate all three drawings on the basis of 14 scoring criteria: arms, attachment of limbs, clothing, ears, eyes, feet, fingers, hair, head, legs, mouth, neck, nose, and torso. Points are awarded for the inclusion of various body parts, the elaboration of the parts, their attachment to each other, their individual and total proportionality, and their location in the drawing. Three separate raw scores from 0 to 64 for all three drawings and a total score can be computed. Total scores can be converted to standard scores and percentile ranks or age equivalents.

The DAP:QSS was normed with a stratified sample of 2622 children 5–17 years old who were representative of 1980 U.S. Census data. The normative sample was stratified for age, sex, race, geographic region, ethnic group, socioeconomic status, and community size. Good psychometric properties such as reliability (i.e., internal reliability coefficients from 0.83 to 0.89 for the total and from 0.56 to 0.78 for the man, woman, and self drawings) and the construct and concurrent validity (i.e., significant correlations with the other measure of nonverbal ability and achievement in reading and math) are reported in the manual for the DAP:QSS [4].

### Raven’s Progressive Matrices

Described as “the paradigm test of nonverbal, abstract reasoning ability” [35, p. 564], Raven’s Progressive Matrices are the oldest, most widely used tests for nonverbal intelligence [36, 37]. They consist of a series of multiple-choice items concerning abstract reasoning of increasing difficulty. Each item presents a logical pattern with a missing element. For each item, participants are asked to identify the correct element from six or eight cells provided below the figure that would best complete the pattern. A participant’s score is the number of correct answers.

In particular, Raven’s Colored Progressive Matrices (RCPM) [38] was designed for individuals in the developmental stage (i.e., 3–12 years of age). It includes 36 items, all colored to attract and maintain children’s attention, divided into three subtests of 12 items each. The maximum possible score for the RCPM is 36. The related Raven’s Standard Progressive Matrices (RSPM) [39] was designed for older children and adults. It includes 60 items in five sets (i.e., A–E), each containing 12 items. The maximum possible score for the RSPM is 60. The RSPM and the RCPM have been regularly evaluated for reliability and validity in various countries all over the world, and they have been shown to be a valid measure of nonverbal cognitive ability [40–44].

To evaluate participants’ performance, the raw scores were compared to recent normative scores (i.e., average scores for age groups and relative centiles), collected during the latest Italian standardization of the RCPM and the RSPM [33, 34].

### Barratt Simplified Measure of Social Status (BSMSS)

The BSMSS [45] is a measure of SES based upon the widely used Hollingshead Four Factor Measure [46] with updated job categories. It provides a simple measure of SES based on marital status, current employment status (or former status for retirees), level of education, and occupational prestige. For school-age individuals, that index is computed as a combination of parents’ educational level and work activity. Occupation was coded in nine groups ranging from 1 (*farm laborers and menial workers*) to 9 (*executives and major professionals*), whereas education was coded in seven levels ranging from 1 (*less than a 7th-grade education*) to 7 (*graduate degree*). Level of education was adjusted to suit the Italian education system. Scores vary from 8 to 66, and higher scores indicate higher SES.

### Statistical Analysis

Cronbach’s alpha (α) was used to assess the homogeneity of DAP:QSS scores. Inter-examiner reliability was determined by calculating the Cohen’s kappa coefficient for the agreement of intelligence classification assigned by each pair of raters to the same drawings in a random sample of 300 drawings. Independent samples *t*-test was carried out in order to investigate group differences in mean scores (boys vs. girls; younger (≤ 11 years old) vs. older (> 11 years old)).

Additionally, Pearson product-moment correlations for bivariate correlations and partial correlations removing effect of SES were conducted to assess the relationships between DAP:QSS score and age, DAP:QSS score and RCPM or RSPM score, and between the DAP:QSS scores and academic grades. Bivariate correlation coefficients were compared using the Fisher *r*-to-*z* transformation. Stepwise multiple regression analyses were conducted to more closely evaluate how well DAP:QSS total score and SES-predicted Raven Progressive Matrices; drawings, Raven Progressive Matrices, and SES scores predicted grades.

The frequency distribution of intelligence classifications according to DAP:QSS, RCPM, and RSPM scores was computed. Because comparable standard scores for the RCPM and RSPM are unavailable, differences between DAP:QSS and RCPM or RSPM scores were assessed by comparing range percentiles with the Wilcoxon signed-rank test for a single sample. Following Willcock et al. [7], to ascertain the effectiveness of the DAP:QSS as a screener of intellectual ability, the scores of children classified as having low or very low intellectual functioning according to the RCPM or RSPM (percentile < 15.5) [33] were compared with their scores on the DAP:QSS for the false negative rate. Conversely, the scores of children identified as having borderline or deficient intellectual functioning according to the DAP:QSS (percentile ≤ 8) [4] were compared with their scores on the RCPM or RSPM for the false positive rate. The concordance between drawings and Raven Progressive Matrices in classification of low intellectual functioning was calculated as sensitivity, specificity, and positive predictive values (PPV) of DAP:QSS total score (implying borderline/deficient intelligence and no borderline/deficient intelligence) and assessed against RCPM/RSPM intelligence classification (indicating low/very low intellectual functioning or no low/very low intellectual functioning). Effect sizes were calculated by means of Cohen’s d for 95%; confidence intervals estimates for the effect sizes were also computed.

Raw scores of variables were used in all analyses, and *p* values < 0.05 were considered to indicate statistical significance. All statistical analyses were performed with the Statistical Package for the Social Sciences version 21.0 for Macintosh.

## Results

### Characteristics of Participants

Twenty-eight schools agreed to participate: nine elementary schools (i.e., four in Naples, one in Avellino, two in Caserta, one in Benevento, and one in Salerno), 11 middle schools (i.e., six in Naples, one in Avellino, two in Caserta, one in Benevento, and one in Salerno), and eight high schools (i.e., four in Naples, one in Avellino, one in Caserta, one in Benevento, and one in Salerno).

Of the 2703 parents who were approached, 2578 consented to allow their children to participate in the study, whereas 125 did not, for a participation rate of 95.37%. Thirty-five parents forgot to read, sign, or return the consent form for their children, 58 children were not given permission from their parents to participate, and 32 children were absent on the day of their test. Of the 2578 participants evaluated, 35 were excluded from analysis: 31 older than 12 years who were erroneously assessed with the RCPM, two for returning incomplete tests that provided RSPM or DAP:QSS data, and two younger than 11 years who were erroneously evaluated with the RSPM. Grades were collected for 894 participants.

The final sample consisted of 2543 participants, mostly of low or middle SES, with the mean grade corresponding to C on the American grading scale. Participants’ characteristics are shown in Table 1.

**Table 1:** Participants’ characteristics (*N* = 2543)

Boys and girls (*n*)	1135 and 1408
Age (years with months)	11.43 (3.06), range: 5.04–17.8
Socioeconomic status	29.25 (11.96), range: 0.0–66
Grades (*n* = 894)	7.68 (1.16), range: 4.58–10.0
Elementary school (*n*, %)	985 (38.73)
Middle school (*n*, %)	854 (33.58)
High school (*n*, %)	704 (27.69)

Data are presented as mean values with standard deviations unless stated otherwise

The reliability analysis (in terms of inter-rater agreement across the four raters) showed Cohen’s kappa coefficients ranging from 0.797 to 0.99.

The Cronbach’s alpha coefficients, means, and standard deviations for each of the three drawings and DAP:QSS total score appear in Table 2, which also shows DAP:QSS scores by the gender and age of participants.

**Table 2:** Cronbach’s alpha (α) coefficients and mean scores (*SD*) for Draw a Person: A Quantitative Scoring System (DAP:QSS scores in the sample (*N* = 2543); drawings’ scores compare boy and girls as well as older and younger participants

DAP:QSS	α	Total sample (*N* = 2543)	Boys *n* = 1135	Girls *n* = 1408	≤ 11 years old *n* = 1039	> 11 years old *n* = 1504
		*M* (*SD*)	*M* (*SD*)	*M* (*SD*)	*M* (*SD*)	*M* (*SD*)
Man	0.789	42.44 (9.7)	41.65 (10.26)	43.07 (9.19)***	38.01 (7.81)	45.49 (9.71)***
Woman	0.749	42.36 (8.71)	41.06 (9.09)	43.4 (8.25)***	37.72 (7.88)	45.56 (7.77)***
Self	0.761	42.97 (8.86)	42.01 (9.27)	43.75 (8.44)***	37.91 (7.81)	46.47 (7.79)***
Total	0.907	127.75 (24.91)	124.7 (26.51)	130.22 (23.27)***	113.6 (22.03)	137.53 (21.92)***

*p < 0*.*05; **p < 0*.*01; ***p < 0*.*001

Girls obtained higher DAP:QSS scores than boys for all drawings (man, *t*(2541) = − 3.620, *p* = 0.000, *d* = − 0.15 [95% CI for effect size: − 0.22, − 0.07]; woman, *t*(2541) = − 6.727, *p* = 0*.*000, *d* = − 0.27 [95% CI for effect size: − 0.35, − 0.19]; self, *t*(2541) = − 4.876, *p* = 0*.*000, *d* = − 0.2 [95% CI for effect size: − 0.28, − 0.12]), as well as total score (*t*(2541) = − 5.511, *p* = 0*.*000, *d* = − 0.22 [95% CI for effect size: − 0.30, − 0.14]). By contrast, RCPM and RSPM mean scores did not differ between boys (RCPM: 26.38 ± 6.34; RSPM: 43.97 ± 8.39) and girls (RCPM: 26.11 ± 6.23; RSPM: 44.56 ± 7.96) in the sample (RCPM *t*(1037) = 0.700, *p* = 0*.*484; RSPM *t*(1502) = − 1.368, *p* = 0*.*172) (data not shown).

### Construct Validity of the DAP:QSS

Children older than 11 years obtained higher raw scores than younger children for all drawings and for the total score (man, *t*(2541) = − 21.469, *p* < 0.001, *d* = − 0.85 [95% CI for effect size: − 0.91, − 0.75]; woman, *t*(2541) = − 24.844, *p* < 0.001, *d* = − 1.01 [95% CI for effect size: − 1.09, − 0.92]; self, *t*(2541) = − 27.235, *p* < 0.001, *d* = − 1.1 [95% CI for effect size: − 1.18, − 1.01]; total, *t*(2541) = − 27.002, *p* < 0.001, *d* = − 1.09 [95% CI for effect size: − 1.17, − 1.01]) (Table 2).

As shown in Table 3, age significantly correlated with DAP:QSS scores across the sample for both boys (total, *r* = 0.526, *p* < 0.0001; man, *r* = 0.430, *p* < 0.001; woman, *r* = 0.483, *p* < 0.001; self, *r* = 0.555, *p* < 0.001) and girls (total, *r* = 0.445, *p* < 0.0001; man, *r* = 0.347, *p* < 0.001; woman, *r* = 0.428, *p* < 0.001; self, *r* = 0.430, *p* < 0.001; data not shown).

**Table 3:** Pearson correlation coefficients of Draw a Person: A Quantitative Scoring System (DAP:QSS) scores and age, DAP:QSS scores (i.e., both bivariate and partial and controlling for SES) and Raven’s Colored Progressive Matrices (RCPM) or Raven’s Standard Progressive Matrices (RSPM), and DAP:QSS scores and grades, all by age

	Total sample					≤ 11-year-olds					> 11-year-olds				
	Age		Grades (*N* = 894)			Age		RCPM (*N* = 1039)			Age			RSPM (*N* = 1504)	
	Bivariate	Partial	Bivariate	Partial		Bivariate	Partial	Bivariate	Partial		Bivariate	Partial		Bivariate	Partial
DAP:QSS man	0.389***	0.383***	0.080*	0.042		461***	0.462***	0.461 ***	0.514 ***		− 0.039	− 0.045		0.156***	0.092
DAP:QSS woman	0.462***	0.453***	0.043	− 0.006		0.454***	0.455***	0.470 ***	0.497 ***		− 0.005	− 0.019		0.174***	0.189***
DAP:QSS self	0.492***	0.486***	0.023	− 0.024		0.479***	0.480***	0.473 ***	0.521 ***		− 0.006	− 0.020		0.178***	0.146**
DAP:QSS total	0.488***	0.481***	0.056	0.007		0.493***	0.494***	0.498***	0.539 ***		− 0.020	− 0.033		0.195***	0.169**

*p < 0.05; **p < 0*.*01; ***p < 0*.*001

However, in an analysis of the sample grouped by age, correlations between DAP:QSS scores and age emerged only for participants aged 11 years or younger but not older ones, even when the effect of SES was considered, as shown in Table 3.

### Concurrent Validity of the DAP:QSS and Its Association with Academic Achievement

DAP:QSS scores were significantly and positively correlated with Raven Matrices scores, with r values ranging from 0.156 to 0.498, even when the effect of SES was removed, with the exception of the association between DAP:QSS score and RSPM score for the drawing of a man, as shown in Table 3. Correlations between DAP:QSS total score and RCPM score were significantly higher (*z* = 8.643, *p* < 0.001) than those between DAP:QSS total score and RSPM score (Table 3).

Across the sample, no correlations were observed between DAP:QSS scores and grades regardless of SES.

In stepwise regression analysis testing DAP:QSS scores and SES as predictors, DAP:QSS total score emerged as a significant predictor of RCPM scores by explaining approximately 24.7% of the variance (*R*^2^ = 0.247, *F* = 338.469, *df* = 1, *p* < 0.001). In the second step of analysis, the addition of SES in the regression equation increased the explained variance by 1.7% (*R*^2^ = 0.263, *F* = 184.615, *df* = 2, *p* < 0.001). In a subsequent stepwise regression analysis testing DAP:QSS scores and SES as predictors, SES surfaced as a significant predictor of RSPM scores by explaining approximately 3.9% of the variance (*R*^2^ = 0.039, *F* = 59.356, *df* = 1, *p* < 0.001). In the second step of that analysis, the addition of DAP:QSS total scores in the regression equation increased the explained variance in RSPM scores by 3.2% (*R*^2^ = 0.071, *F* = 55.850, *df* = 2, *p* < 0.001).

In another stepwise regression analysis testing DAP:QSS scores, RCPM score, and SES as predictors of grades, SES emerged as a significant predictor by explaining approximately 8.8% of the variance (*R*^2^ = 0.088, *F* = 48.986, *df* = 1, *p* < 0.001). In the second step of the analysis, the addition of DAP:QSS scores in the regression equation increased the explained variance by 1.7% (*R*^2^ = 0.105, *F* = 29.611, *df* = 2, *p* < 0.001). Testing DAP:QSS scores, RSPM score, and SES as predictors of grades revealed that RSPM score was a significant predictor (*R*^2^ = 0.23, *F* = 113.786, *df* = 1, *p* < 0.001), as was SES (*R*^2^ = 0.327, Δ*R* = 0.097, *F* = 92.285, *df* = 2, *p* < 0.001) and DAP:QSS total score (*R*^2^ = 0.349, Δ*R* = 0.022, *F* = 67.603, *df* = 3, *p* < 0.001).

### Screening Ability of the DAP:QSS

**Fig. 1:**
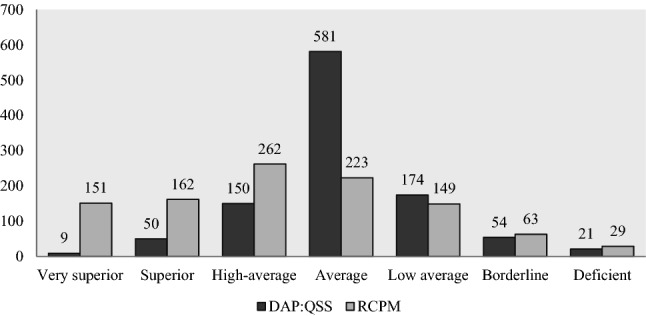
Number of participants classified according to Raven’s Colored Progressive Matrices (RCPM) and Draw a Person: A Quantitative Scoring System (DAP:QSS) scores and related intelligence categories

**Fig. 2:**
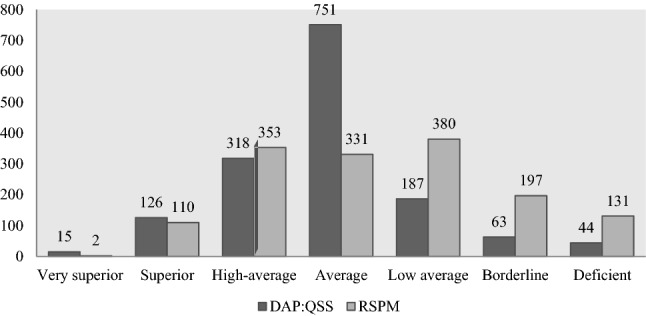
Number of participants classified according to Raven’s Standard Progressive Matrices (RSPM) and Draw a Person: A Quantitative Scoring System (DAP:QSS) scores and related intelligence categories

The number of participants classified according to DAP:QSS and Raven Matrices scores are shown in Fig. 1 (≤ 11 years participants), Fig. 2 (> 11 years participants), and Table 4 (all participants, column Total).

**Table 4:** False negatives (N = 369) and positives (N = 131) of intelligence functioning according to Draw a Person: A Quantitative Scoring System (DAP:QSS) and Raven’s Colored Progressive Matrices (RCPM) or Raven’s Standard Progressive Matrices (RSPM) scores

DAP:QSS score	RCPM or RSPM score							
	Very low	Low	Low average	Average	High average	Superior	Very superior	Total
Deficient	12	6	*15*	*15*	*14*	*2*	*1*	65
Borderline/deficient	19	14	*25*	*21*	*23*	*12*	*3*	117
Low average	**33**	**42**	82	80	73	30	21	361
Average	**78**	**151**	279	292	325	131	76	1332
High average	**14**	**35**	90	101	131	66	31	468
Superior	**4**	**12**	32	39	44	28	17	176
Very superior	**0**	**0**	6	6	5	3	4	24
Total	160	260	529	554	615	272	153	2543

False negatives appear in bold and false positives in italic

A comparison of the percentile ranks obtained by participants on the RCPM or RSPM and the DAP:QSS with the Wilcoxon signed-rank test revealed that scores obtained on the RCPM or RSPM differed significantly from those on the DAP:QSS (RCPM, *z* = − 13.109, *p* < 0.001; RSPM, z = − 10.966, *p* < 0.001).

As shown in Table 4, of the 182 participants indicated to have borderline deficient intelligence or deficient intelligence—all obtained percentile scores of 8.0 or less on the DAP:QSS—82% (*n* = 131 of 182) did not have borderline deficient or deficient intellectual functioning as measured by the RCPM or RSPM, for a high false positive rate. According RCPM and RSPM scores, of those 131 participants, 40 (30.5%) had low average intelligence, 36 (27.4%) had average intelligence, 37 (28.3%) had high average intelligence, 14 (10.7%) had superior intelligence, and four (3.1%) had very superior intelligence (Table 4).

Of the 420 children who obtained percentile scores of 15.5 or less on the RCPM or RSPM, 87.9% (*n* = 369 of 420) were not identified as having borderline intellectual functioning according to the DAP:QSS, for a high false negative rate. The DAP:QSS erroneously evaluated those participants by classifying 75 of 369 (20.3%) as having low average intelligence, 229 (62.1%) as having average intelligence, 49 as having high average intelligence (13.3%), and 16 (4.3%) as having superior intelligence (Table 4).

**Fig. 3:**
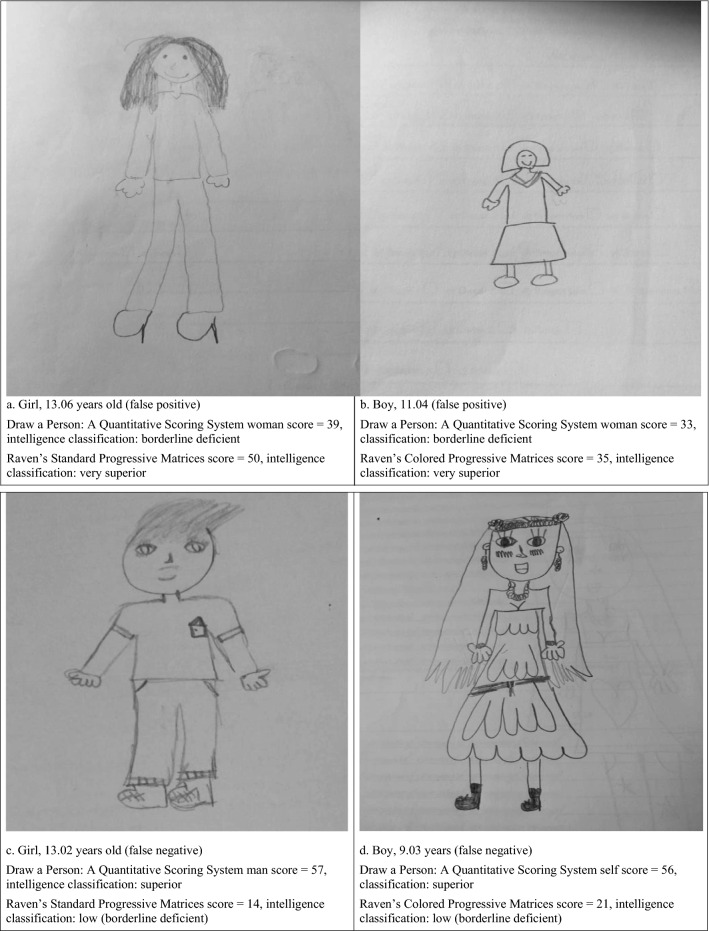
Examples of false positives and negatives

Of the 75 children 11 years old or younger who obtained percentile scores of 8 or less on the DAP:QSS, 73.3% (*n* = 55 of 75) were not of borderline or deficient intellectual functioning as measured by the RCPM or RSPM, for a high false positive rate (Fig. 3, Drawings a and b). Of the 92 children older than 11 years of age who obtained percentile scores of 15.5 or less on the RCPM or RSPM, 78.3% (*n* = 72 of 92) were not identified as having borderline intellectual functioning by the DAP:QSS, for another high false negative rate (Fig. 3, drawings c and d).

For the whole sample, the sensitivity, specificity, and PPV of the DAP:QSS for identifying low intellectual functioning were 0.12, 0.94, and 0.28 respectively.

## Discussion

The study presented here was conducted with a large sample of Italian children (*N* = 2543) across a broad age range, with the aim of examining the psychometric properties of the DAP:QSS. To date, the American norms collected in a sample of 2622 individuals aged from 5 to 17 years have constituted the only available guide for scoring and interpreting Naglieri’s drawings. Therefore, the results obtained provide Italian norms for the DAP:QSS as well.

The present findings support the reliability of the DAP:QSS, in terms of both internal consistency and inter-rater reliability. In particular, Cronbach’s alpha coefficients indicated the good internal consistency for each DAP:QSS drawing, and very good internal consistency for DAP:QSS total score. The internal consistency reliabilities for the single drawings were lower than that of DAP:QSS total score and quite consistent with others reported in the test manual [4].

A significant gender-based effect was also found, which corroborates findings from Saklofske et al. [12] and Schepers et al. [27], as well as normative data. However, it should be noted that Naglieri [4] considered such an effect to be low and lacking sufficient practical significance to require different norms on the basis of gender.

In terms of construct validity, although older children obtained higher raw scores than younger ones for all drawings and for the total score, the findings indicate conflictive evidence for age differentiation: age significantly correlated with DAP:QSS scores only for children 11 years or younger, suggesting that a ceiling effect may have occurred. As affirmed by Scott [19] regarding Goodenough–Harris’s drawing test scores and by Naglieri [4] regarding the DAP:QSS, human figure drawings differentiate performance only between age groups between 5 and 11 or 12 years old when their test scores show a substantial increment associated with increased age. Due to the test’s ceiling effect, no gain in scores could be expected for children older than 12 years of age. That leveling off in scores may be due to the presence of a finite number of items in the drawings for which a typically developed adolescent can readily earn points. Moreover, in early adolescence, the transition from habits of concrete to abstract conceptualization favors increasing distance from material realities and concrete details [19]. Ultimately, the irregularly-changing developmental trend in the drawings’ scores that was found in the present investigation provides inconsistent DAP:QSS construct validity data.

In terms of concurrent validity, the findings indicate positive and significant—albeit modest—correlations between children’s scores on the DAP:QSS and their scores on standard measures of nonverbal intelligence (i.e., RSPM and RCPM) regardless of the effect of SES. In particular, correlation coefficients ranging from 0.156 to 0.498 were comparable and sometimes higher than the correlation levels between the DAP:QSS and analogous measures of nonverbal intelligence (i.e., Matrix Analogies Test-Short Form and RCPM), as reported by previous studies ([4] *r* = 0.19–0.31; [10] *r* = 0*.*32; [11] *r* = 0.30–0.37; [12] *r* = 0.35–0.50; [13] *r* = 0.35–0.50; [15] *r* = 0.35–0.44). Accordingly, a small amount of variance in a child’s Raven’s Matrices scores is predicted by their drawings.

The associations between DAP:QSS and RCPM scores were significantly stronger than the associations between DAP:QSS and RSPM scores. Because the interpretation and features of children’s drawings are confounded by maturational conditions [47], it can be hypothesized that the influence of confounding variables, including artistic ability, low motivation, personal interest in drawing, the degree of adolescent adherence to test instructions, and emotional difficulties frequently experienced during adolescence, could have been played a role in the weak association between adolescents’ drawings and their RSPM performance.

However, as highlighted by Gresham [18], “it does not make much psychometric sense to use human figure drawings to validate the results of an intelligence test when the correlation between the two is between 0 and 0.4, whereas the correlations among intelligence tests is between 0.8 and 0.9” (p. 183). With this in mind, the overall correlations between drawings and Raven matrices described here are substantially low, indicating that the DAP:QSS does not contribute incrementally valid information to intelligence test results.

The DAP:QSS did not correlate significantly with academic achievement and demonstrated very little usefulness in predicting such achievement. Unlike findings presented in the test manual [4] indicating DAP scores correlated significantly with reading and math achievement among students in Grades 4–12 and in other studies [10, 15, 20], those results suggest that a limited relationship exists between DAP:QSS and grades. Such results should be considered to take into account that academic achievement is a product of the dynamic interaction of a hierarchy of factors [48, 49] such that grades (e.g., teachers’ evaluations) may not always accurately reflect general cognitive abilities at the individual level. Even though the results should be interpreted with caution, they should also be recognized as further evidence highlighting that the DAP:QSS is flawed in predicting scholastic performance.

Concerning the usefulness of administering the DAP:QSS to identify children possible at risk of intellectual difficulties, the DAP:QSS was not as effective in screening intellectual ability. Low sensitivity, along with high false positive and negative rates that were quite similar between participants younger and older than 11 years, suggest that the DAP:QSS failed to identify numerous children with intellectual difficulties and falsely identified children with normal and even superior intellectual functioning as having borderline deficient or deficient intelligence. In line with Willcock et al.’s [7] findings, DAP:QSS scores appeared to be of little use as indicators of children’s intellectual functioning.

In sum, the contradictory increase in DAP:QSS mean scores as a function of age, the modest correlations with Raven’s Progressive Matrices, and the lack of significant associations between drawings and grades all clearly reveal the weaknesses in both construct validity and concurrent validity of the DAP:QSS as a measure of general intellectual ability. Moreover, in the light of its inaccuracy in intelligence classification, to the key question of whether it is possible to use the DAP:QSS to identify children who might be at risk of intellectual difficulties, it is possible to answer that decisions about intelligence functioning should never be based upon scores for drawings [5, 15].

Altogether, the study’s results add further support to the research indicating that the human figure drawings—even in the most up-to-date version (e.g., DAP:IQ)—may not be a valid measure of cognitive ability [50, 51].

Several limitations in the study warrant attention. First, the sample, despite its size, was recruited out of convenience and did not include children with mental disabilities. Moreover, the construct validity was examined by analyzing the increase in mean scores as a function of age; therefore, aspects of the construct validity of the DAP:QSS remain to be evaluated, especially discriminant validity. Data of the drawings was interpreted with reference to the original score norms of Naglieri’s drawings. It should be taken into account that DAP:QSS was normed in 1988, and adjustment for the Flynn effects—although observed in human figure drawings [52]—is actually lacking in Naglieri’s drawings and therefore would have been needed for this study. In addition, the assessment of academic achievement was based on grades only, not standardized measures, and performed not as an output in specific subject areas but as a global achievement that might not accurately reflect general cognitive abilities at the individual level.

## Summary

In this study we aimed at assessing the psychometric properties of the DAP:QSS. Although our results produced encouraging evidence of the reliability of Naglieri’s drawings, support for the validity of the drawings as a measure of nonverbal intelligence was rather weak. The DAP:QSS also appeared to be an inaccurate measure of academic performance and ineffective in screening for intellectual ability.

In line with the criticism of psychometric qualities shown by previous and more recent versions of DAP, DAP:QSS consistently failed to produce a psychometrically sound assessment for children’s intellectual functioning. Taken together, this evidence indicates that the utility of human figure drawings as a measure of intelligence is particularly poor, leading to the conclusion that practitioners should not rely on human figure drawing tests as a projective measure of intelligence.

## Data Availability

No prior dissemination of the data and narrative interpretations of the data/research appearing in the manuscript (e.g., data presented at a conference or meeting, posted on a listserv, shared on a website, including academic social networks like ResearchGate, etc.) occurred.
